# Statistical power of phylo-HMM for evolutionarily conserved element detection

**DOI:** 10.1186/1471-2105-8-374

**Published:** 2007-10-05

**Authors:** Xiaodan Fan, Jun Zhu, Eric E Schadt, Jun S Liu

**Affiliations:** 1 Department of Statistics, Harvard University, Boston, MA, USA; 2 Genetics, Rosetta Inpharmatics LLC, a wholly owned subsidiary of Merck & Co., Inc. Seattle, WA, USA

## Abstract

**Background:**

An important goal of comparative genomics is the identification of functional elements through conservation analysis. Phylo-HMM was recently introduced to detect conserved elements based on multiple genome alignments, but the method has not been rigorously evaluated.

**Results:**

We report here a simulation study to investigate the power of phylo-HMM. We show that the power of the phylo-HMM approach depends on many factors, the most important being the number of species-specific genomes used and evolutionary distances between pairs of species. This finding is consistent with results reported by other groups for simpler comparative genomics models. In addition, the conservation ratio of conserved elements and the expected length of the conserved elements are also major factors. In contrast, the influence of the topology and the nucleotide substitution model are relatively minor factors.

**Conclusion:**

Our results provide for general guidelines on how to select the number of genomes and their evolutionary distance in comparative genomics studies, as well as the level of power we can expect under different parameter settings.

## Background

One of the most fundamental problems of molecular biology is to annotate all functional elements in the genome. Because of evolutionary pressure, many of the functional elements are believed to be located within regions that evolve more slowly than the overall genome [1,2]. Regions that evolve more slowly are called evolutionarily conserved elements. Conservation analysis by comparing genomes of related species is a powerful approach for identifying functional elements like protein/RNA coding regions and transcriptional regulatory elements [3-8].

In comparative genomics, one often starts with an alignment of multiple orthologous sequences from several species. The power of a conservation analysis is usually measured by its sensitivity and specificity of detecting conserved elements from given alignments. Several papers have been published recently dealing with the power evaluation of phylogenetic models in comparative genomics studies [9,10]. Both Eddy [9] and McAuliffe et al. [10] evaluated the power of such studies assuming a symmetric star topology and the Jukes-Cantor nucleotide substitution model, whose simple structure makes it possible to evaluate the power of the method analytically. McAuliffe et al. considered the hypothesis testing problem for the conservation of a single nucleotide site, whereas Eddy considered the classification problem for whether a whole DNA block is conserved or not. Both papers reported the theoretical power mainly as a function of the number of comparative genomes and the branch length from the center of the star topology to each species. However, the approaches they employed are not able to accommodate the substitution rate variation and correlations among nearby nucleotides along a genomic sequence. More realistic models are needed to account for the spatial rate variation and correlation in order to more accurately reflect the nature of real data [11]. Various models have been proposed for this task, including the hidden Markov model [12,13], Ising chains [14,15] and Markov random fields [16,17]. In particular, Felsenstein and Churchill [13] used HMMs to model the substitution rate correlation along the genome. Since then, this model has been adapted to many evolution related problems [7,18-20].

In this paper we evaluate the power of the phylogenetic hidden Markov model (phylo-HMM), which models the substitution rate variation using an HMM. As introduced collectively in ref. [7,12,13] to improve phylogenetic modeling, Phylo-HMM is a generative probability model for aligned multiple orthologous sequences. It models the molecular evolution in both the space dimension along the genome and the time dimension along branches of the phylogenetic tree. Along the genome of the common ancestor, an HMM is used to describe the change from one site to the next. Along each branch of the phylogenetic tree, a continuous-time Markov process is used to model the evolutionary process. Siepel et al. [7] implemented a two-state phylo-HMM to perform a genome-wide detection of evolutionarily conserved elements. They showed that this model can yield biologically meaningful results, but did not evaluate the statistical power of the method. In their study, the transition matrix of the HMM in the phylo-HMM model is assumed to be known and unchanged along the genome. This is equivalent to assuming that the expected length and coverage (defined as the percentage) of conserved elements are known and that the conservation ratio is homogeneous along the genome, which is unrealistic [21].

The goal of this paper is to systematically assess the ability (or power) of the phylo-HMM to identify conserved genomic regions. Because this model is too complex for a theoretical analysis and because the availability of real data with experimentally verified conserved elements is still very limited, we adopt a simulation approach. Given that different regions along the genome vary in terms of their neutral substitution rates, the expected lengths and coverage of the conserved elements [21], we used sequence data from promoter regions of four selected species (human, mouse, rat, and dog) to first estimate the set of key parameters of the phylo-HMM. We call this set of estimated parameter values the *baseline*. We simulated sequences by varying one or a subset of parameters around the *baseline*. Using these simulated sequences, we explore several fundamental questions in comparative genomics with respect to this model, including 1) does the topology of the phylogenetic tree critically impact statistical power?, 2) is the accuracy of the predictions significantly reduced if we simplify the nucleotide substitution model?, 3) is it always beneficial to select more distantly related species for this analysis, thereby increasing the branch lengths of the phylogenetic tree?, 4) how many comparative genomes do we need in order to detect conserved elements of a certain length with satisfactory power?, and 5) is spatial variation of the conservation ratio important? Assuming that the underlying phylo-HMM model is a reasonable approximation of reality, our results reveal a number of insights. First, we demonstrate that the most important factors of a comparative genomics analysis appear to include the number of genomes used and the evolutionary distance between pairs of species, consistent with results for simpler models reported by others. Further, we show that the conservation ratio of conserved elements and the expected length of the conserved elements are also major factors. Therefore, our results show that it is important to accurately characterize the inter-species (spatial) variation in the phylo-HMM model. In contrast, the influence of the topology and the nucleotide substitution model are relatively minor.

## Methods

### Phylo-HMM model

**Figure 1:**
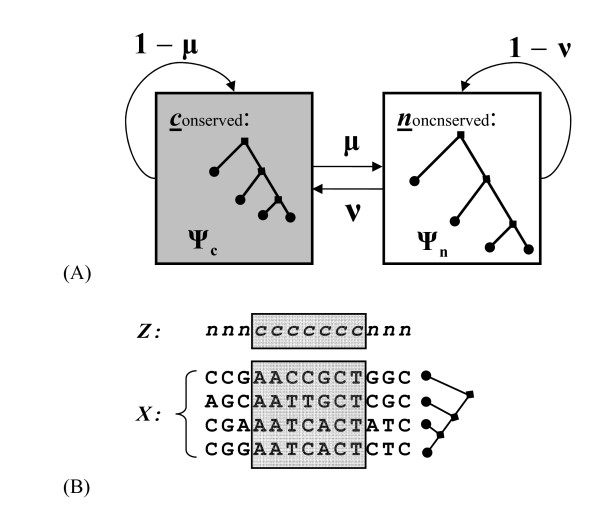
**Two-state phylo-HMM**. (A) State-transition diagram: The system consists of a state for conserved sites (*c*) and a state for nonconserved sites (*n*). The two states are associated with different phylogenetic models (*ψ*_*c *_and *ψ*_*n*_). The state-transition probabilities are defined by two parameters (*μ *and *ν*) as illustrated. (B) An illustrative alignment generated by this model: A state sequence (*Z*) is generated according to *μ *and *ν*. For each site in the state sequence, a nucleotide is generated for the root node in the phylogenetic tree and then for subsequent child node according to the phylogenetic model (*ψ*_*c *_or *ψ*_*n*_). The observed alignment (*X*) is composed of all nucleotides in the leaf nodes. The segment of adjacent conserved sites (in the gray box) is called a conserved element.

A main objective of the conservation analysis is to classify each nucleotide position of the target genome as either evolutionarily conserved or nonconserved based on a comparison with genome sequences of some other chosen species. This task is often achieved by first aligning the genomes under consideration and then using a computational strategy for site classification. Here we focus on the second task. Following the description of the two-state phylo-HMM model (Figure 1) given by Siepel et al. [7], the alignment generating mechanism is modeled as a two-step procedure. First, a common ancestral DNA sequence of all contemporary species under consideration is generated from a two-state HMM, with the hidden states being conserved or nonconserved sites. Second, the phylogenetic model assumes that each nucleotide in the ancestral DNA evolved independently, conditional on their hidden states, to the contemporary nucleotides along all branches of the phylogenetic tree. The two parameters, *(μ, ν)*, for the two-state HMM are derived from the data, and then re-parameterized as *(P, L)*, which have a more intuitive explanation, with *L *= *1/μ *representing the expected length of a conserved element (i.e., a segment of contiguous conserved sites), and *P *= *ν/(μ *+ *ν) *representing the expected coverage of conserved elements (the density of the conserved sites). The phylogenetic models for nonconserved and conserved states are denoted as *ψ*_*n *_= *(Q, π, τ, β) *and *ψ*_*c *_= *(Q, π, τ, ρβ)*, respectively. Here *π *is the vector of background (equilibrium) probabilities for the four nucleotide bases; *τ *is the tree topology of the corresponding phylogeny; *β *is a vector of non-negative real numbers representing branch lengths of the tree, which are measured by the expected number of substitutions per site; *ρ *is the conservation ratio representing the ratio between the substitution rate in conserved regions versus that in nonconserved regions; and *Q *is a 4-by-4 substitution rate matrix for the continuous-time Markov process.

Many parametric forms are available for the substitution rate matrix *Q *[22,23]. Here we used the parametric forms in the PAML package [24]. The JC model [25] is the simplest one among all models implemented in PAML; it assumes a uniform base composition and a uniform rate for all types of substitutions. F81 [26] assumes that the substitution rate is proportional to the frequency of the target nucleotide. HKY [27] is a more realistic rate matrix because it accounts for non-uniform base composition and transition/transversion rate bias. REV, a generalization of HKY, is the most general model and only requires that the nucleotide substitution process be a reversible Markov process [28].

### Parameter inference and posterior probability

We assume that the tree topology *τ *of the selected species for comparative analysis is known. The background distribution *π *can be first estimated by the relative frequencies of the four bases of all the sequences in consideration and treated as known. All other parameters, denoted by *θ *= (*μ, ν, Q, β, ρ*), will be inferred from the data by their maximum likelihood estimates (MLEs). The complete likelihood can be written as:

P(Z,X|θ)=bz1P(x1|ψz1)∏i=2Kazi−1ziP(xi|ψzi)
 MathType@MTEF@5@5@+=feaafiart1ev1aaatCvAUfKttLearuWrP9MDH5MBPbIqV92AaeXatLxBI9gBaebbnrfifHhDYfgasaacH8akY=wiFfYdH8Gipec8Eeeu0xXdbba9frFj0=OqFfea0dXdd9vqai=hGuQ8kuc9pgc9s8qqaq=dirpe0xb9q8qiLsFr0=vr0=vr0dc8meaabaqaciaacaGaaeqabaqabeGadaaakeaacqWGqbaucqGGOaakcqWGAbGwcqGGSaalcqWGybawcqGG8baFiiGacqWF4oqCcqGGPaqkcqGH9aqpcqWGIbGydaWgaaWcbaGaemOEaO3aaSbaaWqaaiabigdaXaqabaaaleqaaOGaemiuaaLaeiikaGIaemiEaG3aaSbaaSqaaiabigdaXaqabaGccqGG8baFcqWFipqEdaWgaaWcbaGaemOEaO3aaSbaaWqaaiabigdaXaqabaaaleqaaOGaeiykaKYaaebCaeaacqWGHbqydaWgaaWcbaGaemOEaO3aaSbaaWqaaiabdMgaPjabgkHiTiabigdaXaqabaWccqWG6bGEdaWgaaadbaGaemyAaKgabeaaaSqabaGccqWGqbaucqGGOaakcqWG4baEdaWgaaWcbaGaemyAaKgabeaakiabcYha8jab=H8a5naaBaaaleaacqWG6bGEdaWgaaadbaGaemyAaKgabeaaaSqabaGccqGGPaqkaSqaaiabdMgaPjabg2da9iabikdaYaqaaiabdUealbqdcqGHpis1aaaa@637B@

where *K *is the total number of columns in the alignment, *x*_*i *_is the observed nucleotide vector in the *i*-th column, *z*_*i *_∈ {*c*, *n*} is the hidden state of the *i*-th column, (*b*_*c*_, *b*_*n*_) = (*ν/(μ *+ *ν*), *μ*/(*μ *+ *ν*)) is the initial emission probability of the HMM, and azi−1zi
 MathType@MTEF@5@5@+=feaafiart1ev1aaatCvAUfKttLearuWrP9MDH5MBPbIqV92AaeXatLxBI9gBaebbnrfifHhDYfgasaacH8akY=wiFfYdH8Gipec8Eeeu0xXdbba9frFj0=OqFfea0dXdd9vqai=hGuQ8kuc9pgc9s8qqaq=dirpe0xb9q8qiLsFr0=vr0=vr0dc8meaabaqaciaacaGaaeqabaqabeGadaaakeaacqWGHbqydaWgaaWcbaGaemOEaO3aaSbaaWqaaiabdMgaPjabgkHiTiabigdaXaqabaWccqWG6bGEdaWgaaadbaGaemyAaKgabeaaaSqabaaaaa@3620@ is the transition probability as illustrated in Figure 1. With the help of the standard forward/backward procedure for HMM [29] to sum over all possible *Z*, we can use the EM algorithm [30] to get the MLE of *θ*, denoted as θ^
 MathType@MTEF@5@5@+=feaafiart1ev1aaatCvAUfKttLearuWrP9MDH5MBPbIqV92AaeXatLxBI9gBaebbnrfifHhDYfgasaacH8akY=wiFfYdH8Gipec8Eeeu0xXdbba9frFj0=OqFfea0dXdd9vqai=hGuQ8kuc9pgc9s8qqaq=dirpe0xb9q8qiLsFr0=vr0=vr0dc8meaabaqaciaacaGaaeqabaqabeGadaaakeaaiiGacuWF4oqCgaqcaaaa@2E79@. Based on θ^
 MathType@MTEF@5@5@+=feaafiart1ev1aaatCvAUfKttLearuWrP9MDH5MBPbIqV92AaeXatLxBI9gBaebbnrfifHhDYfgasaacH8akY=wiFfYdH8Gipec8Eeeu0xXdbba9frFj0=OqFfea0dXdd9vqai=hGuQ8kuc9pgc9s8qqaq=dirpe0xb9q8qiLsFr0=vr0=vr0dc8meaabaqaciaacaGaaeqabaqabeGadaaakeaaiiGacuWF4oqCgaqcaaaa@2E79@, the forward-summation-backward-sampling method [31] can then be used to compute the posterior probability for a given hidden state to be conserved, i.e., *P*(z_*i *_= *c*|*X*, θ^
 MathType@MTEF@5@5@+=feaafiart1ev1aaatCvAUfKttLearuWrP9MDH5MBPbIqV92AaeXatLxBI9gBaebbnrfifHhDYfgasaacH8akY=wiFfYdH8Gipec8Eeeu0xXdbba9frFj0=OqFfea0dXdd9vqai=hGuQ8kuc9pgc9s8qqaq=dirpe0xb9q8qiLsFr0=vr0=vr0dc8meaabaqaciaacaGaaeqabaqabeGadaaakeaaiiGacuWF4oqCgaqcaaaa@2E79@). By applying a threshold to the posterior probability that a given site is conserved, we finally classify all sites in the alignment as either conserved or nonconserved. Another possible approach is to estimate *θ *using a Bayesian method via the Gibbs sampler [31].

### The Baseline

**Figure 2:**
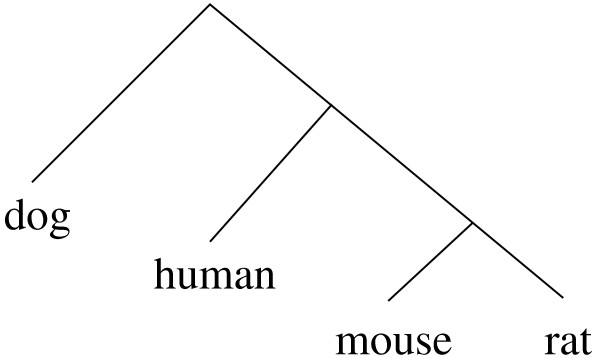
**Topology of the *baseline *phylogenetic tree**. For the un-rooted version of this tree, the branch between the mouse-rat and the human-dog pairs is called the "middle branch".

We estimated the *baseline *parameters of the phylo-HMM from the promoter sequences of dog, human, mouse, and rat, whose evolutionary relationship is represented by the phylogenetic tree in Figure 2. The promoter sequence of a gene is defined as the region 2000 bp upstream to 500 bp downstream of the annotated transcription start site of that gene. MLAGAN [32] was used to align the promoter regions of genes in each *orthologous gene cluster*, which is defined as a set of 4 genes, each from a different species, of which any pair are reciprocal-best BLAST hits of each other (filtered by blastn threshold e-value = 0.001). All gaps in the alignment were treated as missing data [7]. If the average branch length of the phylogenetic tree for the conserved sites of a gene's promoter region was estimated to be greater than one substitution per site, the corresponding promoter alignment was considered unreliable and, thus, removed from our simulation studies. After filtering out such alignments, 8533 orthologous gene clusters remained. Using the REV model for the substitution rate matrix, we fitted the phylo-HMM to each of these 8533 alignments separately. The *baseline *is defined by setting the parameters (*π, μ, ν, Q, β, ρ*) to be the median values of fitted parameters from these 8533 alignments.

### Simulation scheme

Given the phylogenetic tree and all model parameter values (*π, μ, ν, Q, β, ρ*), we first simulated the *true *state sequence from the two-state HMM, considering sequences 2500 nucleotides in length. Then, we simulated the ancestral DNA sequence by independently choosing a nucleotide for each site of the state sequence according to *π*. To simulate the ungapped alignment of the modern-day genomes, we followed the phylogenetic tree from its root for each site. Recursively, we sampled a nucleotide for each branch point (or leaf) of the tree according to the Markov transition kernel *Q *and the length of the branch that links to the previously-generated branch point. Assuming that the true topology and the true substitution model type were known, we computed the posterior probability for each site being conserved from the simulated alignments. The predicted state sequence was produced by applying a threshold to the posterior probability. The sensitivity of the method was defined as the proportion of true conserved sites correctly predicted, and the specificity was defined as the proportion of predicted conserved sites that were truly conserved. The Receiver Operating Characteristic (ROC) curve was plotted to illustrate the tradeoffs between the sensitivity and specificity at different thresholds.

The above simulation-prediction procedure was repeated 1000 times under each topology and parameter setting unless otherwise specified. The median sensitivity and specificity at each threshold were used to draw a ROC curve. The inter-quartile range (i.e., the range from the 1^st ^to 3^rd ^quartiles) was used to reflect the variation of the sensitivity and specificity measures. The size of this range is related to the length of the alignment. We used the median and the inter-quartile range instead of the mean and the standard deviation because the former measures are more robust and naturally bounded by 0 and 1. We used the bootstrap method [33] to obtain the 95% confidence interval of the median. The size of the bootstrap confidence interval is dependent of the number of simulations.

## Results and discussion

### The Baseline

The baseline parameter values for the phylo-HMM with REV, as estimated from alignments of orthologous promoters of dog, mouse, rat, and human, are listed in Table 1. The average length of a conserved element is about 50 bp, much longer than the typical length of a transcription factor binding site. This may be due to the prevalence of the clustering effect of cis-regulatory elements [34], or the existence of sequence features that are specific to individual genes instead of transcription factors. The average coverage of conserved elements is about 25%. Estimated branch lengths of the phylogenetic tree for nonconserved sites show that the promoter region is more divergent compared to the protein coding region. For example, the estimated distance between human and mouse for the promoter region is 0.788 substitutions per site, whereas that for the protein coding region is 0.569 substitutions per site [5].

**Table 1:** The baseline setting

**Parameter**	**Value**				
expected length *L *= *1/μ*:	50				
expected coverage *P *= *ν/μ *+ *ν)*:	0.25				
background probability *π*:	A	C	G	T	
	0.24034	0.25518	0.26097	0.24351	
	A	C	G	T	
substitution rate matrix *Q *(type:REV):	-1.02446	0.23112	0.58662	0.20672	A
	0.21767	-0.99759	0.20665	0.57327	C
	0.54024	0.20206	-0.95781	0.21551	G
	0.20402	0.60073	0.23095	-1.03570	T
un-rooted branch length *β*:	dog	human	mouse	rat	middle
	0.56496	0.33361	0.09604	0.10016	0.35807
conservation ratio *ρ*:	0.32				

The table provides the fitted phylo-HMM parameter values (median values) from the alignments of orthologous promoters of dog, mouse, rat, and human. MLAGAN was used to align the orthologous promoters. Each promoter alignment of the 8533 orthologous gene clusters was fitted separately by phylo-HMM using the REV model for the substitution rate matrix.

### Power comparison of phylo-HMM and the PID method

We simulated alignments from phylo-HMM with the *baseline *setting. These alignments were then used by phylo-HMM to infer the hidden state sequence conditional on the true topology and the true substitution model type, i.e., REV. The power of the phylo-HMM on these simulated alignments was compared with the PID method, which is a simple but widely used local conservation measure in comparative genomics [35-40]. Given an alignment, the PID value of each site was calculated as the percent of identical columns (i.e., completely conserved across all comparative species) within a window centered at the current column. To maximize the performance of the PID method, we set the window size as the true expected length of conserved elements, which is 50 + 1 for the *baseline*. Then the ROC curves were generated by varying thresholds for the PID values.

**Figure 3:**
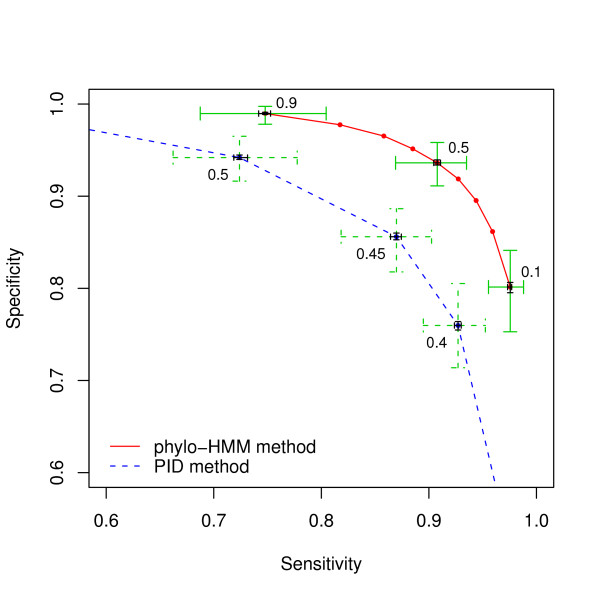
**Power comparison at the baseline**. The red solid line is the ROC curve for the phylo-HMM method. The blue dashed line is the ROC curve for the PID method (window size = 51 bp). The points indicate the median sensitivity and specificity values. The green crosses show their 1^st^-to-3^rd ^quartile range when the threshold is set as the labeled value. The black crosses show the corresponding 95% bootstrap confidence interval of the median sensitivity and specificity.

Both methods were applied to the simulated ungapped alignment under the baseline setting. Figure 3 shows the corresponding ROC curves. By choosing 0.5 as the posterior probability threshold for a site to be conserved, phylo-HMM achieved a median sensitivity of 0.91 at a median specificity level of 0.94. The 1^st ^– to 3^rd^- quartiles of the sensitivity are 0.87 and 0.93, respectively. However, the PID method only achieved a median sensitivity of 0.73 at the same specificity level. The corresponding 1^st ^– to 3^rd^- quartiles of the sensitivity are 0.66 and 0.78, respectively. The inter-quartile range for the estimate is also larger than that of phylo-HMM. The 95% bootstrap confidence interval for the median estimate is very narrow as shown in Figure 3, which implies that the variation of the median estimate is small. Therefore, the power of PID method is significantly smaller than that of phylo-HMM method for the *baseline*. This is generally true if the alignment is generated from the phylo-HMM model with a non-star-topology tree.

### Influence of branch length at different locations in the tree

It has been suggested that the power of a comparative genomics method to distinguish conserved sites from nonconserved ones increases with the total evolutionary distance in the phylogenetic tree [5,41]. However, it is difficult to obtain a reliable orthologous alignment if the genomes under consideration are too divergent. Even if the true orthologous alignment is available, some recent studies have shown that the power decreases with increasing total branch length in some instances [9,10]. Here we investigated the effect of varying branch lengths on phylo-HMM power.

**Figure 4:**
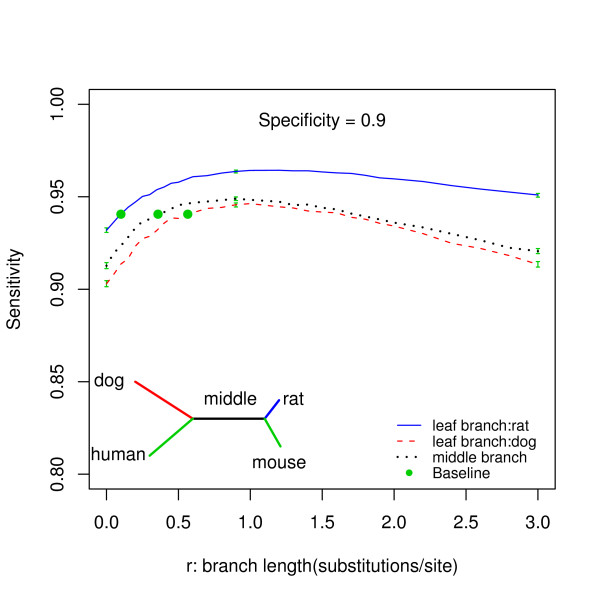
**The relationship between the median sensitivity and branch length at different locations in the tree**. The specificity is fixing at 0.9. The different lines represent the different branches as illustrated in the legend. The green dot indicates the length of the corresponding branch at the baseline. The green whiskers at *r *= 0, 0.9 and 3 indicate the 95% bootstrap confidence interval for the median sensitivity.

The curves in Figure 4, generated by varying lengths of different branches in the baseline phylogenetic tree, show the relationship between the median sensitivity and the length of the branch when the specificity was fixed at 0.9. The simulation-prediction procedure was repeated 10,000 times for each branch length setting considered. It is clear that for the phylo-HMM model, the power decreases if the branch length increases beyond a certain critical value. For example, for the baseline phylogenetic tree, the optimal value for the middle branch is around 0.8 substitutions per site. In addition, the plots show that starting from the baseline branch lengths, the power can be increased more significantly by increasing the length of the shorter branches, rather than increasing the length of the longer branches. This can be seen by noting the way in which the slopes change for all of the curves, where the first derivative decreases as the branch length increases.

### Influence of the tree topology

**Figure 5:**
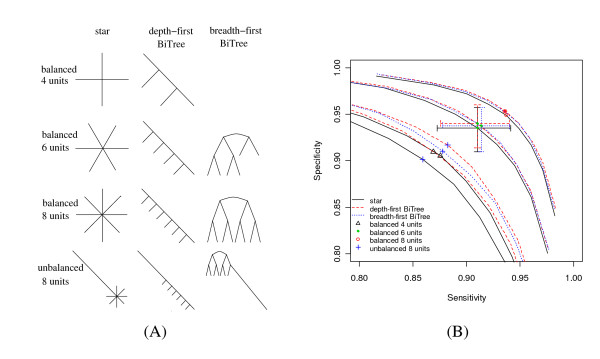
**Power comparison for different topologies**. (A) Topologies considered. Topologies in the same row have the same total branch length and same number of comparative genomes. Each column corresponds to a class of topologies. One unit branch length is 0.196199 substitutions per site (mouse-rat like). The number of comparative genomes is equal to the number of units. For topologies with balanced branch lengths, all branches have the same length. For topologies with unbalanced branch lengths, all branches except the long branch have length equal to 0.02 substitutions per site. The length of the long branch is set to make the total branch length equal to 8 units. (B) Corresponding ROC curves. As illustrated in the legend, the different groups of curves represent different topology classes. Different point groups highlighted along the curves represent the different rows in Figure 5(A). The locations of these points correspond to a posterior probability threshold equal to 0.5. The crosses show the 1^st^-to-3^rd ^quartile range of the sensitivity and specificity.

A common question concerning comparative genomics studies is how to choose the species. One factor to consider is the evolutionary distance, which we have just addressed. Another factor is the topology representing the relationship of these species. By changing the topology and branch length of the baseline model to those in Figure 5A, we performed simulations to compare the power of three classes of representative topologies under different branch length settings (Figure 5B). For topologies with balanced branch lengths, all branches have the same length. For topologies with unbalanced branch lengths, all branches except the long branch have lengths equal to 0.02 substitutions per site. The length of the long branch is set to match the total branch length. When the number of genomes under study was small (e.g., equal to 4), the ROC curve for the symmetric star topology differed very little from the balanced depth-first binary tree. For larger numbers of comparative genomes, the symmetric star topology performed slightly worse than the balanced binary trees. No detectable power difference was observed between the two classes of balanced binary trees.

Figure 5 also shows that the performance of the unbalanced topologies is much worse than the balanced ones. This is because the genomes with very short branches are so close to each other that there is little difference among them. In other words, the clustering effect of these genomes decreases the effective number of genomes in the topology, thus reducing its power. By the same logic, since the clustering effect for the star topology is more serious than for the binary trees, the unbalanced star topology performed much worse than the unbalanced binary trees. The unbalanced depth-first binary tree performed better than the breadth-first one because genomes are more widely dispersed in the depth-first tree. Although not conclusive, these results suggest that the branch lengths should be balanced among all genomes, and if the branch lengths are balanced, the topology is not that important in choosing species for comparative genomics studies. The power of simpler topologies like the symmetric star topology can be used to approximate the power of more complex, but balanced topologies with equal total branch length and the same number of genomes.

### Influence of the number of genomes

**Figure 6:**
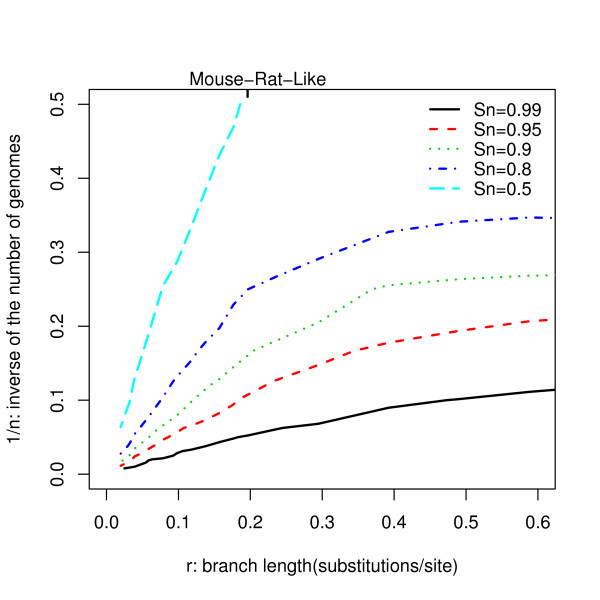
**Influence of the number of genomes for the symmetric star topology**. This graph illustrates the relationship between one over the number of genomes and the individual branch lengths. The specificity is fixed at 0.95 in all cases. Each curve corresponds to a given sensitivity (Sn) level as illustrated in the legend.

The number of species to choose is yet another important problem to consider in performing comparative genomics studies. Based on conclusions from previous sections, we investigated this problem using a symmetric star topology and the common baseline settings, with the exception of the branch lengths (Figure 6). Assuming each branch is as long as the distance between mouse and rat, 6 genomes are needed to achieve a sensitivity of 0.90 at a specificity of 0.95. Adding 4 more genomes increases the sensitivity to 0.95 at this same specificity. For shorter branch lengths (e.g, < 0.2, like the distance between mouse and rat), the number of genomes required to achieve a desired level of power scales inversely with the individual branch length. This scaling property of phylo-HMM is similar to that established for the individual alignment block model [9].

### Effect of different substitution rate models

Several parametric forms are available to model the nucleotide substitution process. Simpler models are obviously easier to handle. For example, the analytical form of the ROC curve is available for the JC model under certain conditions [9,10]. Although models with more free parameters, such as HKY and REV, appear more realistic, conducting a proper statistical inference for these models is difficult, and one can actually lose information or over-fit the data if an improper analysis is done. Therefore, one question is whether we can use simpler models to capture the essential characteristics of the power for the more complex models. We performed two experiments to investigate this problem.

**Figure 7:**
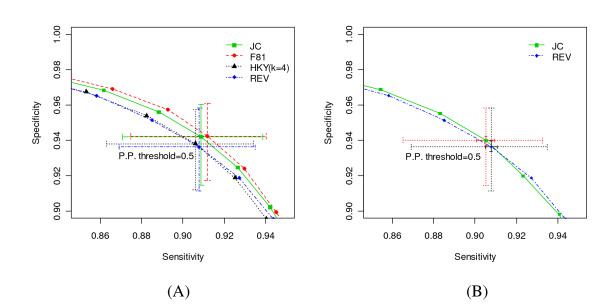
**Influence of substitution model type**. This graph compares the power of phylo-HMM for (A) different substitution model types (JC, F81 with baseline *π*, HKY with kappa = 4 and baseline *π*, REV with baseline *π *and rate matrix), and (B) simulations carried out using the REV model and then estimations carried out using the JC and REV models. The curves again represent the ROC curves as defined in the legend. The crosses highlight points corresponding to the 0.5 posterior probability threshold. The small solid line crosses in (B) show the 95% bootstrap confidence interval of the median sensitivity and specificity. Other crosses show the 1^st^-to-3^rd ^quartile range.

The first experiment involved comparing the power of various model types. To compare them on a common ground, we used the same parameter values as in the *baseline*, with the exception of the substitution rate matrix for each model type. For JC, we used the uniform background nucleotide probability. For HKY, we set kappa = 4. For simulated sequences from each model type, we used the corresponding true model type to infer the state sequence. We went through the simulation-prediction procedure 1000 times to get the ROC curve for each model type. The results are depicted in Figure 7(A) and show that the simpler models (JC and F81) performed slightly better than the more complex models (HKY and REV), which agrees with our intuition that simpler models are easier to solve. At the parameter values set by mimicking these 8533 alignments, however, the observed differences are small.

The second experiment was aimed at characterizing the effects of simplifying the substitution model. The REV model has five free parameters, whereas the JC model has no free parameters. We simulated alignments from the REV model using the baseline parameters, and then inferred the state sequence under the simpler JC model. This perhaps represents a more realistic situation in which the substitution model in the analysis is a simplification of the "true" substitution model. Figure 7(B) shows that the ROC curve for using the true model type is located within the 95% bootstrap confidence interval of the ROC curve for using the simplified JC model, which justifies the use of the simple JC model to study the properties of more complex models such as REV.

### Power comparison for different HMM parameters

Evolutionarily conserved elements within different regions have different sizes and densities. For example, conserved elements in coding regions are much longer than those in promoter regions, and the coverage of conserved elements (i.e., the percentage of sites that are conserved) in coding regions is higher than those in intergenic regions. Also, conserved elements in promoter regions of genes rich in cis-regulatory modules may be longer than those for other genes. Furthermore, promoter regions of different genes may have different coverage of conserved elements. Binding sites for different transcription factors have different lengths as well. All of these variations are modeled by the HMM parameters *μ *and *ν*, or correspondingly, expected coverage (*P*) and expected element length (*L*) in the phylo-HMM method.

**Figure 8:**
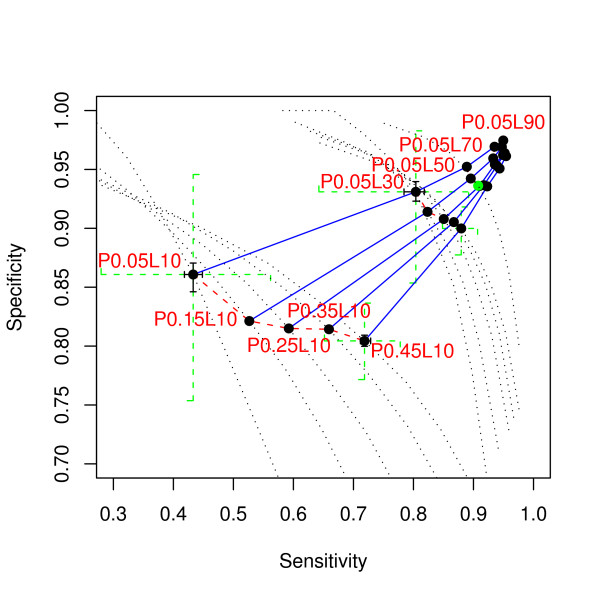
**Power comparison of phylo-HMM for different expected coverage of conserved element (*P*) and expected element length (*L*)**. The black dotted lines are the ROC curves for the *P&L *setting as annotated. For example "*P*0.05*L*10" means *P *= 0.05 and *L *= 10. The points are their power at posterior probability threshold equal to 0.5. The point corresponding to the baseline (P0.25L50) is indicated as a green dot. The blue solid lines connect the points with the same *P*, while the red dashed line connects the points with the same *L*. Some of the points are highlighted by crosses. The green dotted line crosses show the 1^st^-to-3^rd ^quartile range. The black solid line crosses show the 95% bootstrap confidence interval of the median sensitivity and specificity.

We simulated alignments by varying the expected coverage and length of conserved elements in order to understand their effect on the power of phylo-HMM (Figure 8). Under the same expected coverage, a greater expected element length yields better ROC curves with smaller variation. For example, for the same expected coverage *P *= 0.25, to achieve a median specificity of 0.94, the median sensitivity for expected element length *L *= 10, 30, 50, 70, 90 is 0.30, 0.80, 0.91, 0.94, and 0.97, respectively. The relationship between the median sensitivity and *1/L *is approximately linear with a negative slope. The effect of the expected coverage for fixed expected element length is rather complicated because the corresponding ROC curves cross over. For example, when the expected element length *L *= 30, to achieve a low specificity like 0.8, the sensitivity for expected coverage *P *= 0.45 is higher than that for *P *= 0.05. To achieve a high specificity like 0.95, the sensitivity for expected coverage *P *= 0.45 is lower than that for *P *= 0.05. The variation of the ROC curves for smaller expected coverage is greater than for bigger ones in the range we considered (*P *= 0.05, 0.15, 0.25, 0.35, 0.45). The area under the ROC curve of a larger expected coverage is greater than that of a smaller expected coverage. In this sense, a larger expected coverage of conserved elements makes them easier to detect.

### Power comparison for different conservation ratio

**Figure 9:**
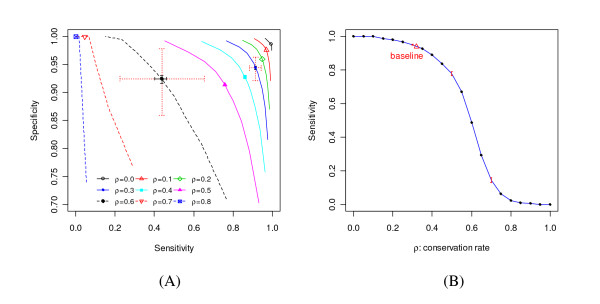
**Power comparison of phylo-HMM for different conservation ratio (*ρ*)**. (A) Comparing the whole ROC curves. The ROC curves shown are for different *ρ *as illustrated in the legend. The locations of the points correspond to a posterior probability threshold equal to 0.5. Some of the points are highlighted by crosses. The red dotted line crosses show the 1^st^-to-3^rd ^quartile range. The black solid line crosses show the 95% bootstrap confidence interval of the median sensitivity and specificity. (B) The relationship between sensitivity and the conservation ratio at a fixed specificity equal to 0.9. The dots are where power was evaluated by simulation. The red whiskers at *ρ *= 0.3, 0.5, and *ρ *= 0.7 indicate the 95% bootstrap confidence interval for the median sensitivity. The red triangle indicates the power of the *baseline*, where *ρ *= 0.32.

The conservation ratio (*ρ*), which is defined as the ratio of the average substitution rate of conserved sites over that of nonconserved sites, is one of the major factors determining the power of phylo-HMM. Figure 9 shows the power we can expect for a given conservation ratio under other baseline settings. For fixed specificity, the relationship between sensitivity and the conservation ratio is approximately a sigmoid type function: 1/(1 + *e*^13(*ρ*-0.6)^). The power decreases dramatically with increasing conservation ratio, especially when the conservation ratio is around 0.6. This poses a problem for the two-state phylo-HMM model, which assumes a uniform conservation ratio along a given alignment. A promoter region could be bound by several types of transcription factors, and each of these could have a different conservation ratio compared to the nonconserved background. In this case, the power evaluation from a uniform conservation ratio is questionable. This problem can be alleviated by introducing multiple rate classes to form a multiple-state phylo-HMM.

### Ability to recover the true alignment and its influence on the power

All simulations discussed so far have been based on the assumption that we can get the true alignment. Therefore, the usefulness of the above results is questionable in more realistic situations where the true alignment is unknown. We again used simulation to check what alignment accuracy we could achieve under different phylo-HMM parameter settings. We simulated sequences that were 2500 base pairs long by varying one or a subset of phylo-HMM parameters around their baseline values. We then used MLAGAN [32] to align the simulated sequences, assuming that we knew the topology of the true evolutionary tree. Real alignment problems are plagued by many different types of noise in the input sequences (e.g., insertions and deletions), which inevitably decreases the alignment accuracy. In order to evaluate the alignment accuracy determined by phylo-HMM parameters, we directly fed the sequences simulated based on the phylo-HMM to MLAGAN without adding additional insertions or deletions. The alignment accuracy was measured by the column score [42], which is defined as the number of identical columns between the true alignment and the recovered alignment. We further divided the number of identical columns by 2500 to normalize the column score to the [0, 1] interval.

**Figure 10:**
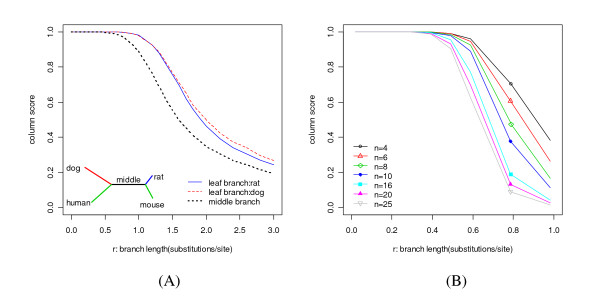
**The relationship between the column score and branch length**. (A) Relationships for branches at different locations in the baseline phylogenetic tree. The different lines represent the different branches as illustrated in the legend. (B) Relationships for branches in the symmetric star-topology tree. The different lines correspond to the different numbers of genomes (n) represented by the tree.

We evaluated the alignment accuracy for all situations reported in the previous sections. The column score was higher than 0.99 for the baseline case and in all cases where the topology, substitution rate model, HMM parameters, and the conservation ratio were varied. Figure 10 shows the influence of branch length and the number of genomes. For the baseline phylogenetic tree, the column score decreases below 0.99 if any of the leaf branches is longer than 1 substitution per site or the middle branch is longer than 0.6 substitutions per site. For the symmetric star-topology tree, if the number of genomes is 4, the column score decreases below 0.99 if the single branch length is longer than 0.5 substitutions per site. As the number of genomes increases, the column score decreases below 0.99 at shorter branch lengths, which implies it is harder to recover the true alignment if the number of genomes increases. All of these results suggest that no branch length should be longer than 1 substitution per site (e.g., the distance between dog and rat) in order to realize an accurate alignment. If no branch is too long, which holds around the baseline setting, the assumption that a highly accurate alignment is available is valid.

**Figure 11:**
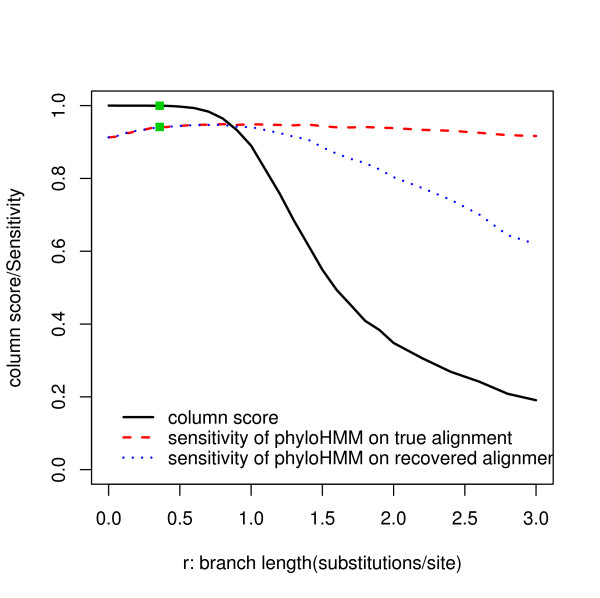
**The influence of alignment quality to the power of phylo-HMM**. Simulations were done by varying the length of the middle branch in the baseline phylogenetic tree. The black solid line shows the relationship between the branch length and the column score. The red and blue dashed lines show the relationships between the branch length and the median sensitivity for the true and the recovered alignments, respectively, with the specificity fixed at 0.9.

To further investigate the influence of alignment quality on power, we fitted phylo-HMM to both the true alignment and the recovered alignment. Previously we defined sensitivity and specificity by comparing the true and predicted state sequences. Since there is no unique true state sequence corresponding to the recovered alignment, we instead counted the number of nucleotides located within conserved sites. We call these nucleotides as conserved nucleotides. Sensitivity is defined as the proportion of true conserved nucleotides correctly predicted. Specificity is defined as the proportion of predicted conserved nucleotides that were truly conserved. For the true alignment, these definitions are equivalent to the previous version defined by conserved sites. Figure 11 shows the performance of phylo-HMM on the true alignment and the recovered alignment when we varied the length of the middle branch in the baseline phylogenetic tree. When the column score is no less than 0.98, the difference of the median sensitivity between the two scenarios is less than 0.001 when the specificity is fixed at 0.9. When the column score decreases to 0.6, this difference increases to 0.046, which is still quite small. This suggests that the power of phylo-HMM is robust to the alignment quality measured by column score. In the case of changing middle branch length in the baseline phylogenetic tree, the noise caused by bad alignment begins to pull down the power when the branch length grows to be greater than one substitution per site.

Besides MLAGAN, we also evaluated the alignment accuracy using TBA [43] and MAVID [44], which have been used for the recently published ENCODE consortium alignments [45]. Details are shown in additional file 1. Among these three aligners, TBA is essentially a local alignment algorithm, which avoids incorporating distant sequences into the output alignment. MAVID produces global alignments by assuming that all sites in the alignment evolve at the same speed. MLAGAN produces global alignments for sequences containing conserved blocks, which is the exact scenario we are dealing with. The alignment accuracies are similar for the results of the three aligners when the branch lengths are short (See supplementary Figures 1 and 2 in additional file 1). These results agree with those reported by Kumar and Filipski [46]. Nevertheless, the power of phylo-HMM is still quite robust with respect to the alignment quality.

## Conclusion

We used simulations to investigate the statistical power of phylo-HMM under a number of diverse situations. Among all factors studied, the number of species-specific genomes used, evolutionary distance, conservation ratio, and expected length of the conserved element are the major factors affecting the power of phylo-HMM. If conditions allow, it is better to select species such that every branch length in the phylogenetic tree is between 0.6 to 1 substitutions per site. To achieve a desired power, the number of genomes required in the analysis scales inversely with the mean branch length if the mean branch length is small. The conservation ratio and the expected length of the conserved element are uncontrollable factors. For a fixed specificity, the relationship between the median sensitivity and one over the expected length of the conserved element is approximately linear with a negative slope. We also found that the influence of the topology and the nucleotide substitution model were relatively minor. This justifies selecting species with a simpler topology, like the symmetric star topology, and approximating complex substitution models with less complex, easier to manipulate ones, like the JC model.

Our analyses of the power of phylo-HMM models were carried out under a number of simplifying assumptions that may influence the results reported and the degree to which they will reflect what can be expected from real data. First, for the simulations carried out to evaluate power, we assumed that all sequences were generated from the phylo-HMM model and that we could get the true ungapped alignment. In reality, it may be difficult to locate the orthologous sequences. Even if we can get the orthologous alignment, gaps are inevitable and it is perhaps inappropriate to treat them as missing data. Second, the real nucleotide substitution process may be more complicated than the models we studied here. For example, content dependent substitution is possible. Finally, the assumption of two evolutionary rates, one for functional elements and the other for neutral background, is also unrealistic and may affect the power estimate we obtained. However, these issues notwithstanding, the general guidelines established by our analysis should still hold qualitatively.

Several problems are worthy of further study. First, a goodness-of-fit test for phylo-HMM on real data remains to be validated. Second, in the current phylo-HMM model, once the conservation state of a site is determined for the common ancestor, it is fixed for all species and is not allowed to change over the course of evolution. More flexible models that allow for differences among the conservation states are required for different species. Third, the conservation annotation conducted by the current phylo-HMM model represents just one step toward the goal of functional annotation. A more ambitious approach would be to directly model the functional elements like transcription factor binding sites. An integrative approach to align the sequences by modeling evolution events and perform the conservation analysis at the same time is also desired. This may be feasible within the start topology JC framework.

## Authors' contributions

XF conceived and carried out the study. All authors participated in designing the study and preparing of the manuscript.

## Supplementary Material

Additional file 1 XFan_PowerOfPhyloHMM_manuscript_v12_supplement.doc. Performance of three Aligners (MLAGAN, TBA and MAVID). It gives detail results for the performance of three aligners. Click here for file
