# Supplementary material for: Statistical power of phylo-HMM for evolutionarily conserved element detection
Source: BMC Bioinformatics. 2007 Oct 5;8:374. doi: 10.1186/1471-2105-8-374 (PMC2194792; doi:10.1186/1471-2105-8-374)
Supplement: Additional file 1 — XFan_PowerOfPhyloHMM_manuscript_v12_supplement.doc. Performance of three Aligners (MLAGAN, TBA and MAVID). It gives detail results for the performance of three aligners. [file 1471-2105-8-374-S1.doc]

**Additional file 1.**

**Performance of three Aligners (MLAGAN, TBA and MAVID)**

The major power analysis results in the manuscript are based on the true alignment, which do not depend on any specific aligner. MLAGAN was used to generate multiple sequence alignments from the real data for estimating baseline parameters, and subsequently used to demonstrate that there exists an aligner that can align the simulated sequences well enough for the power analysis conclusion to hold so long as no branch is too long. Of course, each aligner has its own strengths and is designed for different tasks, and the main aim of this paper was not to compare different aligners using simulated sequences. Nevertheless, we did perform the alignment performance evaluation for the two other aligners (TBA & MAVID). We have redrawn Figure 10 here:

**Supplementary Figures 1 (Corresponding to Figure 10) - The relationship between the column score and branch length**

(A) For branches at different locations in the baseline phylogenetic tree. The different lines represent the different branches as illustrated in the legend. (B) For branches in the symmetric star-topology tree.

For cases where all branch lengths are less than 0.5 substitutions per site, the performance of TBA is similar to that of MLAGAN. However, if a branch is longer than 0.5, TBA performed significantly worse than MLAGAN. This most likely is due to the fact that MLAGAN is a global alignment program, whose output includes all nucleotides in the given sequences. In contrast, TBA (based on blastz) is essentially a local alignment program, whose output does not include all input nucleotides. In particular, when a branch is too long, TBA will choose to not incorporate sequences at the corresponding branch into the final alignments, therefore the corresponding column score is much lower.

The performance of MAVID is much worse than TBA and MLAGAN. TBA and MLAGAN are designed to take advantage of the existence of highly conserved seeds (the conserved elements in the simulated sequences from phylo-HMM). MAVID assumes all sites in the alignment evolve at the same speed, so it is unfair to compare MAVID with TBA and MLAGAN with respect to aligning sequences simulated from phylo-HMM. This point is noted in a recent aligner comparison paper [1], where it is shown that the performance of LAGAN (pairwise version of MLAGAN) is better than Blastz (pairwise version of TBA) and much better than ClustalW (similar to MAVID) if there exists conserved blocks.

We also checked the influence of TBA and MAVID alignment quality on the power evaluation. Because TBA (as a local alignment program) doesn't include all input nucleotides in the output alignment, we cannot compare the output alignment with the true alignment for power evaluation. We are only able to perform this on MAVID. We have redrawn Figure 11 to show the results here:

**Supplementary Figures 2 (Corresponding to Figure 11) - The influence of alignment quality to the power of phylo-HMM**

Simulations were done by varying the length of the middle branch in the baseline phylogenetic tree. The black solid line shows the relationship between the branch length and the column score. The red and blue dashed lines show the relationships between the branch length and the median sensitivity for the true and the recovered alignments, respectively, with the specificity fixed at 0.9.

The power of phylo-HMM is seen to be robust with respect to the alignment quality measured by column score. Again, this graph also shows that the alignment accuracy of MAVID is much worse than that of MLAGAN in this scenario. But we can still say that the sensitivity difference between the inferred and true alignments is quite small when the branch lengths are less than 0.6 and the specificity is equal to 0.9.

In conclusion, under the conditions used in this manuscript, MLAGAN can generate alignments similar to the true alignments and the phylo-HMM power analysis is not sensitive to the alignment difference. The results are similar for TBA and MAVID when branch lengths are short.

**References:**

1. Kumar S, Filipski A: **Multiple sequence alignment: in pursuit of homologous DNA positions.** *Genome Res* 2007 **17**:127–135.
